# Supplementary material for: GC-MS and LC-MS Pesticide Analysis of Black Teas Originating from Sri Lanka, Iran, Turkey, and India
Source: Toxics. 2022 Dec 30;11(1):34. doi: 10.3390/toxics11010034 (PMC9862525; doi:10.3390/toxics11010034)
Supplement: Supplementary file 1 [file toxics-11-00034-s001.zip › toxics-2089039-supplementary.pdf]

Table S1. List of pesticide compounds analyzed by LC-MS / MS in tea

4-D, 2.4-DB, 2-2.Naphthyloxyacetic acid, 3,4,5-Trimethacarb, 3-Hydroxycarbofuran, 4-CPA5-Hydroxy-clethodim-sulfone, 5-Hydroxy-imidacloprid, 5-Hydroxy-thiabendazol, 6-Chlor-3-phenyl-pyridazin-4-ol (Pyridate-Metabolit), Acephate, Acequinocyl, Acetamiprid, Acetochlor, Acibenzolar-S-methyl, Acifluorfen,Aclonifen,Acrinathrin, Alachlor, Aldicarb-sulfoxid, Aldoxycarb, Alloxydim, Ametryn, Amidosulfuron, Aminocarb, Aminopyralid, Amitraz, Amitrol, AMPA (Aminomethyl phosphonic acid), Anilazine, Anilofos, Aramite, Atrazin, Atrazine-2-hydroxy, Atrazine-desethyl, Atrazine-desethyl-2-hydroxy, Atrazine-desisopropyl, Avermectin B1a, Azaconazole, Azamethiophos, Azimsulfuron, Azinphos-ethyl, Azinphos-methyl, Azoxystrobin, Beflubutamid, Benalaxyl, Benazolin, Bendiocarb, Benfuracarb, Benfuresate, Benomyl, Benoxacor, Bensulfuron-methyl, Bensulide, Bensultap, Bentazone, Benzoximate, Bifenazate, Bifenox, Bifenthrin, Bioresmethrin, Bitertanol, Boscalid, Brodifacoum, Bromacil, Bromadiolone, Bromophos, Bromophos-ethyl, Bromoxynil, Bromuconazole, Bupirimate, Buprofezin, Butafenacil, Butocarboxim-sulfoxid, Butoxycarboxim, Butralin, Buturon, Butylate, Cadusafos, Carbaryl, Carbendazim, Carbetamide, Carbofuran, Carbosulfan, Carboxin, Carfentrazoneethyl, Carpropamid, Cartap, Chinomethionate, Chlorbromuron, Chlorbufam, Chlorethoxyfos-oxon, Chlorfenvinphos, Chlorfluazuron, Chloridazon, Chlorimuron-ethyl, Chlormephos, Chlorophacinone, Chlorotoluron, Chloroxuron, Chlorpropham, Chlorpyrifos, Chlorpyrifos-methyl, Chlorsulfuron, Chlorthiamid, Chlorthiophos, Cinerin, ICinerin, IICinidon-ethyl, Cinosulfuron, Clethodim, Clethodim-imin-sulfone, Clethodim-imin-sulfoxide, Clethodim-sulfone, Clethodim-sulfoxide, Clodinafop-propargyl, Clofentezine, Clomazone, Clomeprop, Clopyralid, Cloquintocet-mexyl, Clothianidin, Coumaphos, Coumatetralyl, Crotoxyphos, Cyanazine, Cyanofenphos, Cyanophos, Cyazofamid, Cyclanilide, Cycloate, Cycloxydim, Cyfluthrin, Cyhalofop-butyl, Cymoxanil, Cypermethrin, Cyphenothrin, Cyproconazole, Cyprodinil, Cyromazine, Daminozide, Deltamethrin, Demeton-S-methyl, Demeton-S-methyl-sulfon, Desmedipham, Desmethyl-formamido-pirimicarb, Desmethyl-pirimicarb, Desmetryne, Dialifos, Di-allate, Diazinon, Dichlofenthion, Dichlofluanid, Dichlorprop-P, Dichlorvos, Diclobutrazol, Diclofop-methyl, Dicloran, Diclosulam, Dicrotophos, Dicyclanil, Diethofencarb, Difenacoum, Difenoconazole, Difenoxuron, Difenzoquat, Diflubenzuron, Diflufenican, Diflufenzopyr, Dimefuron, Dimepiperate, Dimethachlor, Dimethametryn, Dimethenamide, Dimethoate, Dimethomorph, Dimetilan, Dimoxystrobin, Diniconazole, Dinoseb, Dinoterb, Dioxathion, Diphacinone, Diphenamid, Diphenylamine, Diquat, Dithianon, Dithiopyr, Diuron, DNOC, Dodemorph, Dodine, Edifenphos, Endosulfan, EPN,

Epoxiconazole, EPTC, Esfenvalerate, Ethametsulfuron-methyl, Ethidimuron, Ethiofencarb, Ethiofencarb-sulfon, Ethiofencarb- Fenamidone, Fenamiphos, Fenarimol, Fenazaquin, Fenbuconazole, Fenfuram, Fenhexamid, Fenitrothion, Fenobucarb, Fenoprop, Fenothiocarb, Fenoxaprop-ethyl, Fenoxycarb, Fenpiclonil, Fenpropathrin, Fenpropidin, Fenpropimorph, Fenpyroximate, Fenthion, Fentin, Fenuron, Fenvalerate, Fipronil, Fipronil-desulfinyl, Fipronil-sulfide, Fipronil-sulfone, Flamprop-M-isopropyl, Flamprop-M-methyl, Flazasulfuron, Florasulam, Fluazifop (free acid), Fluazifopbutyl, Fluazinam, Flucycloxuron, Flucythrinate, Fludioxonil, Flufenacet, Flufenoxuron, Flumetsulam, Flumioxazin, Fluometuron, Fluoroglycofene-ethyl, Fluoxastrobin, Flupyrsulfuron-methyl, Fluquinconazole, Flurenol, Flurochloridone, Fluroxypyr, Fluroxypyr-meptyl, Flurprimidole, Flurtamone, Flusilazole, Flusulfamide, Fluthiacet methyl, Flutolanil, Flutriafol Fluxofenim, Fomesafen, Fonofos, Foramsulfuron, Formetanate, Fosthiazate, Fuberidazole, Furalaxyl, Furathiocarb, Glufosinate, Halfenprox, Halofenozide, Halosulfuron-methyl, Haloxyfop-etotyl, Haloxyfop-P, Haloxyfop-P-methyl, Heptenophos, Hexaflumuron, Hexazinone, Hexythiazox, Hydramethylnon Imazalil, Imazamethabenz-methyl, Imazapic, Imazapyr, Imazaquin, Imazosulfuron, Imibenconazole, Imidacloprid, Imidacloprid-Olefin, Indoxacarb, Iodosulfuron-methyl sodium, Ioxynil, Iprobenfos, Iprodione, Iprovalicarb, Isazofos, Isofenphos, Isofenphos-oxonIsoprocarb, Isoprothiolane, IsoproturonIsoxaben Isoxadifen-ethylIsoxaflutole, Isoxathion, Jasmolin, IJasmolin IIKresoxim-methyllambda-Cyhalothrin, Lenacil, Linuron, Lufenuron, Malaoxon, Malathion, Maleic hydrazide MCPA, MCPA-2-Ethylhexylester, MCPA-butotyl, MCPB, Mecarbam, Mecoprop-P, Mefenacet, Mefenpyr-diethyl, Mepanipyrim, Mepiquat, Mepronil, Mesosulfuron-methyl, Mesotrione, Metalaxyl, Metamitron, Metazachlor, Metconazole, Methabenzthiazuron, Methacrifos, Methamidophos, Methfuroxam, Methidathion, Methiocarb, Methiocarb-sulfoxid, Methomyl, Methomyl-oxime, Methoxyfenozide, Metobromuron, Metolachlor, Metolcarb, Metosulam, Metoxuron, Metrafenone, Metribuzin, Metsulfuron-methyl, Mevinphos, Molinate, Monocrotophos, Monolinuron, Monuron, Myclobutanil, Naled, Napropamide, Neburon, Nicarbazin (1,3- N,N'-bis (4-nitrophenyl)urea), Nicosulfuron, Nicotine, Nitenpyram, Norflurazon, Norflurazon-desmethyl, Novaluron, Nuarimol, Ofurace, Omethoate, Orbencarb, Oxadiargyl, Oxadiazon, Oxadixyl, Oxamyl, Oxamyl-oxime, Oxasulfuron, Oxycarboxin, Oxydemeton-methyl, Oxyfluorfen, Paclobutrazol, Paraoxon, Paraoxon-methyl, Parathion, Parathion-methyl, Pebulate, Penconazole, Pencycuron, Pendimethalin, Permethrin, Pethoxamid, Phenmedipham, Phenthoate, Phorate, Phorat-sulfonPhorat-sulfoxide, Phosalone, Phosmet, Phosphamidon, Phoxim, Picolinafen, Picoxystrobin, Piperonyl butoxide, Piperophos, Pirimicarb, Pirimiphos-ethyl, Pirimiphos-

methyl, Primisulfuron-methyl, Prochloraz, Procymidone, Profenofos, Prohexadione, Promecarb, Prometon, Prometryne, Propachlor, Propamocarb, Propanil, Propaquizafop, Propargite, Propazin-2-hydroxy, Propazine, Propetamphos, Propham, Propiconazole, Propoxur, Propoxycarbazone sodium, Propyzamide, Prosulfocarb, Prosulfuron, Prothioconazole, Prothioconazole, Desthiometabolit, Prothiofos, Pymetrozine, Pyraclofos, Pyraclostrobin, Pyraflufen-ethyl, Pyrazophos, Pyrethrin, IPyrethrin, IIPyridaben, Pyridaphenthion, Pyridate, Pyrifenox, Pyrimethanil, Pyriproxyfen, Pyroquilon, Quinalphos, Quinmerac, Quinoclamine, Quinoxyfen, Quizalofop-ethyl, Quizalofop-P (free acid),Resmethrin, Rimsulfuron, Rotenone, Sebuthylazine, Sebuthylazine-desethyl, Sethoxydim, Siduron, Silthiofam, Simazine, Simazine-2-hydroxy, Simetryn, Spinosyn, ASpinosyn DSpiroxamine, Sulcotrione, Sulfentrazone, Sulfometuron-methyl, Sulfosulfuron, Sulfotep, Sulprofostau-Fluvalinate, Tebuconazol, Tebufenozide, Tebufenpyrad, Tebupirimfos, Tebutam, Tebuthiuron, Teflubenzuron, Temephos TEPP , Tepraloxydim, Terbumeton, Terbuthylazine, Terbuthylazine-2-hydroxy, Terbuthylazine-desethyl, Terbutryn, Tetrachlorvinphos, Tetraconazole, Tetramethrin, Thiabendazole, Thiacloprid, Thiamethoxam, Thidiazuron, Thifensulfuron-methyl, Thiobencarb, Thiodicarb, Thiofanox-sulfone, Thiofanox-sulfoxide, Thiophanate, Thiophanate-methyl, Tolclofos-methyl, Tolylfluanid, Tralkoxydim, Triadimefon, Triadimenol, Tri-allate, Triasulfuron, Triazamate, Triazophos, Triazoxide, Tribenuron-methylTrichlorfon, Triclopyr, Tricyclazole, Tridemorph, Trietazine, Trifloxystrobin, Triflumizole, Triflumuron, Triflusulfuron-methyl, Trinexapac-ethyl, Triticonazole, Tritosulfuron, Uniconazole, Vamidothion, Warfarin, Ziram

Table S2. List of pesticide compounds analyzed by GC-MS in tea

2-Ptenyphenol, 2-keto ethofurnesate, 3.5-Dichloroanline, Acrinathrin, Aldrin and Dieldrin, Benfluralin, Bifenazate, Bifenox, Bifenthrin, Biphenyl, Boscalid, Bromophos-ethly, Bromophos-methyl, Bromoprepylate Bupirimate, Buprofezin, Butylate, Cadusafos, Captan, Carbosulfan ,Carboxin, Chlorbenside, Chlorbufam, Chlorrdane, Chlorethoxyfos, Chlorfenapyr Ott Chloropropylate methyl, Chlorfenvinphos, Chlormephos, Chlorobenzilate, Chloroneb, Chloropropylate, Chlorothalonil, Chlorpyrifos-ethyl, Chlorpyrifos –rnethyl, Chlorthal-dimethyl, Chlorthiamid, Chlozolinate, Clothianidin, Cyanofenphos, Cyflufenamid, Cypermethrin, Cypermethrin, DDT, Dialifos, Diazinon,Dichlobenil, Dichlorvos (DDVP), Dicofol, Diethofencarb, Dimethipin, Diniconazole, Dinobuton, Dioxabenzofos, Diphenamed, Diphenylamine, Disulfoton (sum of disulphoton, disulfoton sulfoxide and disulphoton sulfone; in disulphoton), Ditalimfos , Endosulfan (endosulfan-sulphate with alpha and beta isomers; as endosulfan), Endrin, Endrin Ketone, Ethalfluralin, Ethion, Ethofumasate, Ethoprophos, Etoxazole, Etridiazole, Etrimfos, Fenamidone, Fenarimol, Fenbuconazole, Fenchlorphos (sum of fenchlorphos and fenchlorphos oxon; in phenchlorphos), Fenitrothion, Fenoxaprop-P-ethyl, Fenoxaprop-ethyl, Fenpropathrin, Fenson. Fenvalerate (Any ratio of isomers containing Esfenvalerate (RR, SS, RS and SR)), Flucythrinate, Flurmetralin. Filuridone, Flusilazole. Flutriafol, Folpet (Folpet and Phtalimide sum; in folpet),Fonofos, Formothion, Fosthiazate, Halfenprox, Heptachlor (sum of heptachlor and heptachlor epoxide, in heptachlor), Hexachlorobenzene(HCB), Hexachlorociclohexane(HCH) (sum of isomers excluding gamma isomer) Hexaconazde, Imazalil, lodofenphos (Jodfenphos), Iprodione, Isodrin, Isofenphos, Kresoxim-methyl, Lampda-Cyhalothrin, Lindane (Hexachlorociclohexane (HCH) gamma isomer), Mefenpyr-diethyl, Methacrifos, Methidathion, Methoxychlor, Metribuzin, Mirex, Nitrofen, Oxdiazon, oxsadixyl, Oxyfluorfen, Parathion ethyl, (Parathion), Parathion-methyl (Sum of parathion-methyl and paraoxon-methyl in terms of Parathion-methyl), Penconazole, Pendimethalin, Pentachloroaniline, Pentachloroanisole, Pentanochlor, Permethrin (sum of isomers), Pethoxamid, Phorate, Phorate Sulfone, Phosmed, Phosphamidon, Procmidone, Profenofos, Propachlor, Propazine, Pyrazophos, Pyridaben, Pyrimethanil, Pyrimidifen, Quinoxifen, Quintozone (PCNB) (sum of quintozene and pentachloroaniline in quintozene), Sulfotep, Tebuconazole, Tebufenpyrad, Tebupirimfos, Tecnazene, Tefluthrn, Terbacil, Terbutryn, Tetrachlorvinphos, Tetraconazole, Tetradifon, Tetrasul, Thiocyclam. Thiometon, Tolclofos-methyl, Triazophos, Tribufos, Trichloronat, Triflumizole, Trifuralin, Triticonazole, Vinclozalin, p,p-dichlorobenzocphenone
